# Endorsing Sustainable Enterprises Among Promising Entrepreneurs: A Comparative Study of Factor-Driven Economy and Efficiency-Driven Economy

**DOI:** 10.3389/fpsyg.2021.735127

**Published:** 2021-11-25

**Authors:** Ghulam Raza Sargani, Yuansheng Jiang, Deyi Zhou, Abbas Ali Chandio, Mudassir Hussain, Nawab Khan

**Affiliations:** ^1^College of Economics, Sichuan Agricultural University, Chengdu, China; ^2^School of Economics and Management, Huazhong Agricultural University, Wuhan, China; ^3^Department of Education and Research, University of Lakki Marwat KPK, Lakki Marwat, Pakistan; ^4^College of Management, Sichuan Agricultural University, Chengdu, China

**Keywords:** theory of planned behavior (TPB), PLS-SEM (partial least squares structural equation model), entrepreneurial intention (EI), sustainable enterprises, sustainable development goals—SDGs, work values creation, China and Pakistan

## Abstract

Understanding business trails among promising aspirants may contribute to an actual motive for diminishing ecological tracks and escort to developing devotion toward deciding intentions across various entrepreneurial types and tiers solely from the sustainability domains. Therefore, this study endeavors to comprehend and seek to employ the Theory of Planned Behavior (TPB) to inspect the relationship between antecedents on sustainable enterprise intention and sustainable value creation. In this study, we used the convenience sampling method and the quantitative data of 1,070 respondents from Pakistan and China and applied a SmartPLS structural equation model and partial least square path modeling by mediational and multigroup analyses. Findings divulge that R^2^ (79.8%) value in the Pakistan sample of attitudes to sustainability, perceived entrepreneurial desirability (PED), and perceived entrepreneurial feasibility (PEF) was comparatively higher than in China. The R^2^ (75.6%) variance value on sustainable entrepreneurial intentions (SEI) was recorded higher in the Pakistani sample. However, the relationship of environmental values, self-efficacy, and extrinsic and intrinsic rewards show positive and significant mediational effects on both the economies of SEI. The findings disclosed an inconsistent character of extrinsic rewards, general self-efficacy, and job security depict negative significant impacts of aspirations on sustainable entrepreneurship (SE) among promising entrepreneurs on sustainability enterprises in both Pakistan and China. This study extends on existing entrepreneurship literature. Results supported the designed hypothesis and played a significant role in shedding light on an individual trait underpinning a career in a sustainable business start-up. The study looks at the issue from the viewpoint of sustainability domains. It seeks to determine the individual PED, PEF, and attitude toward sustainable entrepreneurship (ATSE) as the mediational variables. The study highlights the importance of work values in pursuing sustainability-oriented entrepreneurship programs for promising aspirants to improve their entrepreneurial skills and knowledge podium, which will encourage them to become sustainable future entrepreneurs. Furthermore, the study provides understandings for ratifying sustainable openings and debates the potential paths for sustainable business growth and opportunities among nascent entrepreneurs in both economies.

## Introduction

In the face of rising concerns about global warming, climate change, and its severe environmental effects, sustainability entrepreneurs are recognized widely as forging ahead. In many contemporary debates, sustainable businesses primarily focus on meeting basic needs while reducing human environmental footprints on product markets (Sher et al., 2020). Therefore, it is necessary to recognize the innovative practices of sustainable enterprises in sharing professional knowledge so that the fledgling entrepreneurs can improve productivity, quality, and market value, to meet the aspirations and goals of the rural society (Sargani et al., 2020). The fundamental notions of sustainable entrepreneurship (SE) and innovation ethics serve as pillars for furthering corporate solutions to address the most unrelenting social and environmental issues confronting the leading industries in the future (Sargani et al., 2020). On the contrary, sustainable enterprises provide access to inputs and information on eco-friendly management of natural resources, such as improving soil fertility, water conservation, and integrated pest management practices, thus motivating others to pursue their ideas and solutions (Sargani et al., 2020). Nevertheless, activists and academics argue that entrepreneurship can help combat climate change, reduce erosion and environmental scarcity, increase freshwater supplies and agricultural productivity, preserve biodiversity, and conserve habitats (Dean and McMullen, 2007; Ben Youssef et al., 2018). These efforts will significantly impact the developed and developing countries, boosting their sustainable economy, productivity, entrepreneurship opportunities, self-reliance, and socioeconomic stability in the long run to achieve sustainable development goals (SDGs) (Muñoz and Cohen, 2018).

Sustainable entrepreneurship, being the most diverse area of reality, has grown in popularity over the last few decades, representing the advantages, climate, social culture, and economic complexities of business solutions, and demonstrating the growth of businesses powered by function earnings (Stubbs, 2017). In the context of norms, more models and theories proposed so far are entirely driven by mature knowledge fields, especially environmental economics and social entrepreneurship (Shepherd and Patzelt, 2014; Dees, 2018). When entrepreneurship practices lead the vital notions behind SE activity, they explore business growth and economic prospects that undermine the processes of social and ecological arenas (Schaefer et al., 2015). However, this environment that promotes a balance between social, natural, and economic activities must be nurtured or restored for future generations (Parrish, 2010; Schaefer et al., 2015). As the main body of the economy, sustainable entrepreneurs are essentially full of dynamic potential, but they do not create many profits. They seek to start, adjust, and integrate these processes and activities to develop sustainable, driven, profitable economic opportunities, and carry out fundamental changes (Lans et al., 2014). Numerous scholars define sustainable entrepreneurs as individual economic representatives of an enterprise that integrate social, economic, and environmental entrepreneurial aims, and these monetary aspects should be sustainable in terms of wealth creation and business goals (Tilley and Young, 2006). Though SE has been recorded, such as in “Our Common Future,” it fosters a vital understanding of this new realm of entrepreneurship that can incorporate social, environmental, and economic value creation to ensure the long-term well-being of the society with classic work as the distinct knowledge source for environmental and social entrepreneurship (Hockerts, 2017). Similarly, Shepherd and Patzelt (2014) believed that entrepreneurial activities play a significant role in maintaining ecosystems while delivering lucrative benefits (both economic and noneconomic) for entrepreneurs, stockholders, and society doing SE.

On the social level, entrepreneurship contributes to job creation and economic development (Zahra and Wright, 2016). SE, on the other hand, is crucial to achieving critical SDGs, such as public health, market innovation, industrial technology, sustainable cities, balanced production and consumption, and climate change adaptation, which all enable grassroot players to engage in community welfare and social values (Apostolopoulos et al., 2018; Perez Alonso et al., 2018). The basic types of entrepreneurship in accelerating entrepreneurship appear over time to solve the potential social problems (Rai et al., 2017; Sargani et al., 2020). Therefore, the attention of the people has been raised to recognize the intention and potential motivation of becoming such an entrepreneur among the educated youths of the future (Sargani et al., 2020).

Hence the values in SE combine the essence of social, economic, and ecological value creation in a longer time frame (Hockerts, 2015). Similarly, several scholars have suggested a connection between these principles and SE, with innovativeness and economic benefits gaining equal heft (Gibbs, 2009). In this way, some are more inclined to this type of entrepreneurship and actively believe that some entrepreneurial countries are more attractive than others, mainly because of their huge differences in structure and value preferences (Baron, 2008). However, despite the more significant development of entrepreneurship and the remarkable transformation from ancient enterprises to modern innovativeness, there is a lack of evidence to show the unique role of motivation and different values in specific forms of entrepreneurs (Liñán and Fayolle, 2015; Sargani et al., 2020). Furthermore, there has been limited evidence of the inclination in type-specific entrepreneurial activity driving its acceptance, particularly in the case of developing nations (Liñán and Fayolle, 2015; Sargani et al., 2020).

The Y millennials/young generation and notable alumni at the universities of today, represent their strong entrepreneurial aspirations, social consciousness, and focus on youth growth, integration, and well-being; as well as the enormous potential to engage in such demanding activities without compromising their prospects (Hewlett et al., 2009; Sargani et al., 2020). Despite the rising momentum in start-ups, there is little evidence to support the entrepreneurial intention of multiple start-ups (Sargani et al., 2020). To this aim, an important and relevant issue arises: to accelerate the entrepreneurial process, educated young people are more interested in learning about the intentions and underlying motives of prospective entrepreneurs. Moreover, the youths’ intentions are at the center of recognizing opportunities, and critical to understand and envisage entrepreneurial prospects; the intention to have or build a business or adapt an existing one in line with SDGs (Figge et al., 2002; Schaltegger et al., 2016). To realize scientific debates on how environmental values influence sustainable entrepreneurship (Weidinger, 2014) and which need to be based on environmental values, i.e., eco-friendly and green values (Cervelló-Royo et al., 2020).

The concept, “entrepreneurship” has emanated in the corporate world to define this most entrepreneurial and business-driven emphasis on long-term creative business solutions to support society (Schaltegger, 2018). When addressing environmental, economic, and social problems by entrepreneurial practices, the literature lacks a crucial definition of sustainable enterprise, especially in different countries, such as Pakistan and China, which face the same problem of below the average and stagnant SE (Sargani et al., 2020). To address this, policymakers in such countries have attempted to boost SE and effectively involve youths to cater to new career paths; preference perceptions and intentions must be seen as key elements in refining the sustainable entrepreneurial intentions (SEI) (Liñán and Fayolle, 2015; Rosique-Blasco et al., 2018). Pakistan and China were selected in the present study because of the differences between the two economies due to SE bottom lines. China has an emerging and efficiency-driven economy, growing more robust worldwide; However, Pakistan is factor-driven, and the developing economy faces several challenges and many setbacks (Munir et al., 2019; Sargani et al., 2020). Numerous countries, like Pakistan, benefit from the rapid economic development of China, either directly or indirectly. Both the countries collaborate in China–Pakistan Economic Corridor (CPEC) with the young millennial engagement for work and study. Given the fact that both nations collaborate in a range of fields and employ students for jobs and study, a joint knowledge of value creations and their effect on sustainable entrepreneurship (SE) may provide vital outcomes from which both countries may benefit (Munir et al., 2019; Sargani et al., 2020).

Therefore, scholarly emphasis on sustainable development is increasing rapidly, and researchers address the field from a broader perspective to encourage entrepreneurship with SE as a major driver in predicting enterprises activities (Lourenço, 2013). It is important to have entrepreneurial skills to identify opportunities that lead to sustainable growth (Hall et al., 2010). Because of this, entrepreneurs are seen as the engines of sustainable development (Shepherd and Patzelt, 2011), and their aptitude to innovate plays a key role in securing a more sustainable future. Considering the academic debate, numerous scholars assume that entrepreneurs can respond to the demands of the well-being of society (Hall et al., 2010). Due to its increasing prominence and a greater deal of attention, entrepreneurship must be originated on environmental principles to achieve sustainability practices. However, prevailing entrepreneurship intentional models ground an inquiry gap and are inept at answering the type-specific entrepreneurial research and practice from a sustainability perspective. Therefore, comparative and cross-cultural evidence has not yet been debated in these domains to predict SE behavior. In the current state of knowledge, this study explores the motives of start-up intentions among the Chinese and Pakistani aspirants in SE. As such, within the framework of the theory of planned behavior (TPB), i.e., perceived entrepreneurial desirability (PED), perceived entrepreneurial feasibility PEF, and attitude towards sustainable entrepreneurship (ATSE) and General self-efficacy (GSE), job security (JSEC), intrinsic rewards (INTR) extrinsic (EXTR) rewards, on antecedents to cater for their role through an individual’s start-up intentions. In particular, this study aimed to adapt and expand current entrepreneurial intention models in a cross-country context by including work values and attitudes toward sustainable entrepreneurship.

## Research Backgrounds

### Theoretic Context of Sustainability-Oriented Entrepreneurship

A broad frame of literature has undertaken in-depth research on an entrepreneurial motive since the inception of the theory of entrepreneurial event (TEE) (Shapero and Sokol, 1982) and TPB models resulting in the following five research themes: the primary entrepreneurial intention model, the relationship between entrepreneurial behavior and intention, the determinants of cultural, institutional, and regional levels, entrepreneurship education, and social and sustainable-oriented entrepreneurship and setting of the industry entrepreneurship education background (Liñán and Fayolle, 2015).

In the light of the study by Dentchev et al. (2018), it is noted that the advent of this trend is primarily social entrepreneurship, particularly SE. As time passes, less attention is paid to sustainable enterprises. This research has discovered a strong link between entrepreneurial intention and sustainable growth among promising students (Hockerts et al., 2018; Muñoz and Kimmitt, 2019).

Similarly, in the research by Hall et al. (2010), it was observed that the trend toward sustainability is favorably linked to emerging markets and new concepts that reduce environmental footprint. As a result of research into the role of education in the advancement of entrepreneurship, participation in the innovation process and the development of sustainable enterprises are positively linked to shared identity and self-efficacy, which may then be affected by education *via* active management and the sharing of knowledge in SE (Weidinger, 2014; Bux and van Vuuren, 2019). Moreover, results from the study of Demirel et al. (2019) showed that ecological values, such as external and intrinsic rewards, self-efficacy, and social support, are the driving forces influencing the willingness of enterprises to fascinate sustainably.

Although the substantial contribution of Shepherd and Patzelt (2014) and Sher et al. (2020) revealed SE and sustainable agribusiness development, the current investigation has intended to discover one or scarcely two planes of sustainable value creation. The study adapted from Sargani et al. (2020) introduced driving factors of SEI and attitudes toward sustainable agriculture entrepreneurship to extend the prior models into sustainability-oriented entrepreneurship.

However, when coping with the unique sense of SE, the term, value creation at work, for example, environmental and social values, can vary from the conventional term (Burnett et al., 2007). Eco-friendly entrepreneurship is focused on creating environmental value, and social entrepreneurship is closely linked to a culture that values social value creation (Cohen et al., 2019). Furthermore, economic value development has become an integral part of conventional entrepreneurship, in which entrepreneurs engage in various activities to combine multiple economic values (Liñán and Fayolle, 2015).

**FIGURE 1:**
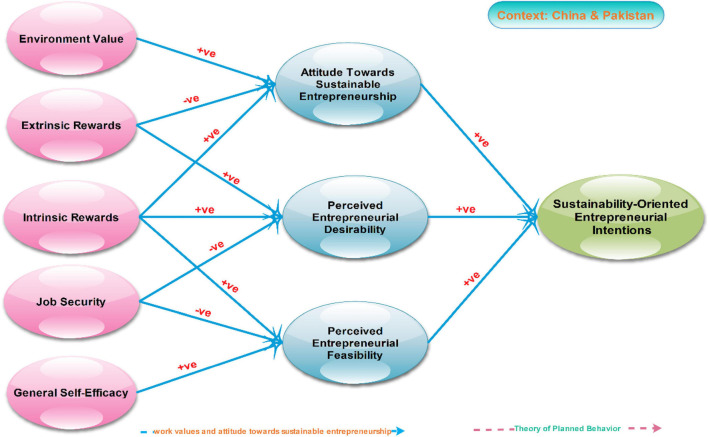
The research framework of the study based on hypothesized constructs adopted form Sher et al. (2020) and Vuorio et al. (2018).

The investigation of Schaltegger and Wagner (2011) and (Schaltegger and Wagner, 2011; Fayolle and Gailly, 2015) showed that self-efficacy, entrepreneurial feasibility, and perceived behavior control put forward the perception phenomenon attitude of completing the same tasks and is therefore treated equally. Adapting and introducing drivers (factors) of SEI) and ATSE expand the previous TPB model into sustainability-driven entrepreneurship. As a result, constructions on environment value, intrinsic reward, ATS, and SEI explicitly incorporate social and environmental value creation dimensions (Figure 1). By adding these variables, the present model includes ecological and social value creation. In contrast, PEF and PED are implicit reflections of economic value creation. Supplemented by general self-efficacy, PEF, and perceived behavioral control have all been linked to consider the driving factors of the SEI phenomenon.

### Sustainability-Oriented Entrepreneurship Proxies

As previously mentioned, two of the points in favor of SE include supplying youth with a diverse range of job options and identifying and evaluating opportunities (Shepherd and Patzelt, 2014; Hanohov and Baldacchino, 2018). As the findings of Koe and Majid (2014) showed, the youth seek the adequacy of their perceived feasible knowledge, abilities, and skills and their perceived desire to pursue specific opportunities for possible career choices (Fitzsimmons and Douglas, 2011). The PEF, which refers to the belief range of individuals, possesses the knowledge, ability, and skills needed to become entrepreneurs or perform challenging tasks; that is, PED indicates how specific entrepreneurial careers are attractive. Simultaneously, prior empirical literature points out that SEI has a positive relationship with the early stage of TPB, such as ATSE, PEF, and PED (Liñán and Santos, 2007; Guerrero et al., 2008; Sher et al., 2020).

### Self-Efficacy and Work Values of Individuals in Sustainable Entrepreneurship

Since job values reflect work success and inspiration, they provide a different way to support, inspire, propel, and foster individual entrepreneurial activity. This propensity is closely linked to the attitude of the former toward a specific profession (Kirkley, 2016). It is worth noting that the work values consider the obvious and not apparent results that a person can achieve by pursuing a job or a business undertaking (Swaney et al., 2012). These perceived work goals and work choices are formed by work values called external and internal rewards (Vansteenkiste and Ryan, 2013). Therefore, an external reward of a person refers to the attraction of the person to personal interests. This kind of value pursues a job to obtain more power, prestige, money, reward, and status (Antonioli et al., 2016).

On the contrary, intrinsic rewards describe learning, innovation orientation, and problem-solving behaviors, encouraging individuals to seek innovative skills, new opportunities, and solve challenging problems (Twenge et al., 2012; Sargani et al., 2019). At present, the research on social entrepreneurship has taken environmental value and job security as the other two work values (Fukukawa et al., 2007; Twenge et al., 2012). Environmental values describe the altruistic behavior of a person (e.g., universalism, compassion, and altruism), which leads to caring for individuals, the surrounding environment, and other individuals in the society with enthusiasm and passion (Lyons et al., 2010). Also, unlike environmental values, safety defines personal preferences for security, stability, and harmony in the workplace (Twenge et al., 2012). According to Patzelt and Shepherd (2016), entrepreneurial career choice is positively related to risk-taking and negatively related to work safety. Likewise, in the studies by Barbosa et al. (2007) and Monteiro et al. (2019), individuals with higher entrepreneurial career choices tend to value security less and are more independent and risk-takers. Therefore, both external and internal rewards are positive, while a sense of security is negatively correlated with higher entrepreneurial intention and has a direct positive correlation with internal reward and SEI. However, this study scrutinizes the role of four fundamental work values (environmental values, sense of security, external, and internal rewards), because these values are highly related to different forms and levels of entrepreneurship and different forms of value creation. Hence ecological values and intrinsic rewards are positively correlated with environmental and social value creation, while the external reward is positively correlated with economic value creation.

In essence, work values are an essential link for individuals to choose work through attitude and motivation (Swaney et al., 2012). Precisely, driving forces and values guide individuals to choose a particular form of the decision-making process. The decision to engage in a particular job or work to become an entrepreneur is more likely to be different from others and may take numerous methods (Jaén and Liñán, 2013). Since work values guide the process of opportunity identification and evaluation through ideal behaviors and goals, there is a clear link between the attractiveness of specific entrepreneurial choices, individual PED, and work values (Esfandiar et al., 2019). Whereas the main motive of pursuing external value is the monetary benefit, prestige, and status, the extrinsic reward is positively related to SEI) and PED (Choo and Wong, 2006; Antonioli et al., 2016). Therefore, if adequately followed, SE also offers higher income, social status, and esteem (York and Venkataraman, 2010; Schaltegger and Wagner, 2011; Sher et al., 2020). Based on the given literature on work values viz-á-viz innovativeness, wealth and power creation, and entrepreneurial pledge and attention, the following relationships are established between the studied work values, such as extrinsic reward, intrinsic reward, and job security with PED and SEI.

Similarly, environmental importance is closely linked to a positive mindset and plays a critical role in advancing SE and shaping and driving focus of an individual on the workable value of social and ecological facets of creation in search of various opportunities (Weisgram et al., 2011; Swaney et al., 2012). Current studies have mainly focused on two facets of value creation: environmental and economic (Dean and McMullen, 2007; Hanohov and Baldacchino, 2018; Domurath and Patzelt, 2019) and generally lack social value creation in SEI, even though social value creation is critical to the appetite of an individual for social entrepreneurship (Wilson and Post, 2013). In comparison, environmental importance and intrinsic reward are the motivators for people to solve social issues.

Sustainable entrepreneurship connects the environmental, economic, and social values (Cohen et al., 2019; Douglas et al., 2020), whereas intrinsic rewards, conjointly with prosocial motivations, play significant uprightness in creating, recognizing, evaluating, and exploiting business opportunities with environmental and societal problems (Shepherd, 2015). Furthermore, the study indicates that environmental importance increases the probability of sighted opportunities by altruistic actions (e.g., altruism and empathy) and PED in ecological and social entrepreneurship (Shepherd and Patzelt, 2014; Sher et al., 2020).

Conferring to a study by Hockerts (2015), SE is solely focused on identifying entrepreneurial opportunities that restore the disequilibria caused by a disparity of the usage of natural resources by converting industries into more socially and environmentally acceptable norms. Though the study Sargani et al. (2020) revealed that SE provides triple value creation, combining disparate values to generate economic value will clash with goals for creating environmental and social values. Extrinsic rewards, in particular, fuel entrepreneurship; the literature indicates that a preference for norms and authority is negatively linked to environmental attitudes (Fukukawa et al., 2007; Twenge et al., 2012). Thus, ATSE has a negative relationship between extrinsic reward and SEI, while intrinsic reward positively correlates with SEI. According to the literature (Muñoz and Cohen, 2018), since environmental and social challenges are complex, the existing structural strategies frequently seem unrealistic. Nevertheless, these uncertainties, socioeconomic difficulties, and social capital building can be overcome by ingenuity and proactive ATSE (Shukla, 2021), which is confirmed by the study and theoretical basis on the relationship among external rewards, intrinsic reward, and ATSE with SEI.

Likewise, great arguments relate to entrepreneurial career choice with sovereign innovativeness risk-taking (Hessels et al., 2008; Bolton, 2012). As a result, it is proposed that a presumed entrepreneurial interest corresponds to the recognition of an individual with regard to a particular entrepreneurial performance. The livelihood of the choice of a person appears to enable him to pursue the following personal ideals linked to the apparent accomplishments of the profession (Segal et al., 2005). Furthermore, a previous study has shown that human risk-taking, independence, individuality, and creativity are linked to entrepreneurial career engagement (Hessels et al., 2008). Since risk-taking is a prerequisite for entrepreneurs to consider, autonomy and creativity are directly linked to an entrepreneurial profession. Job security seems to be negatively associated with PEF and SEI. This reveals that PEF is associated with work security and internal compensation and that internal reward is connected to SEI.

On the other hand, individual self-efficacy is described as a vision capacity applied to various entrepreneurial contexts. It is related to the degree of self-efficacy (Zimmerman, 2000). When looking for demanding tasks, people with higher self-efficacy are more likely to prefer self-employment (Pihie, 2009; Piperopoulos and Dimov, 2015). Similarly, confident people with their abilities, talents, and competence level stick to a single company, which decides whether the business succeeds or fails (Hechavarria et al., 2012). Self-efficacy and personal PEF are inextricably linked. PEF shows a degree of confidence in the people so that they can begin and complete a particular task (Krueger et al., 2000; Fitzsimmons and Douglas, 2011). According to research, it is found that individuals with higher self-efficacyare more confident in their ability to achieve their goals. As a result, self-efficacy is linked to being an entrepreneur and the likelihood of being a profitable entrepreneur (Arrighetti et al., 2016). The study by Smith and Woodworth (2012) revealed the critical mechanism of forming the intention to uphold social value entrepreneurship and shows a connection between self-efficacy and SE.

In the light of the above theoretical mechanism and pieces of evidence, the following hypotheses are designed for the current study:

### Research Hypotheses

**H1.** Attitude toward SE will positively mediate the relationship between environment value and SEI among Chinese as well as Pakistani aspirants.
**H2.** Attitude toward SE will positively mediate the relationship between intrinsic reward and SEI among Chinese as well as Pakistani aspirants.
**H3.** Attitude toward SE will negatively mediate the relationship between extrinsic reward and sustainability-driven entrepreneurial intentions among Chinese as well as Pakistani aspirants.
**H4.** Perceived entrepreneurial desirability will positively mediate the relationship between extrinsic reward and SEI among Chinese as well as Pakistani aspirants.
**H5.** Perceived entrepreneurial desirability will positively mediate the relationship between intrinsic reward and SEI among Chinese as well as Pakistani aspirants.
**H6.** Perceived entrepreneurial desirability will negatively mediate the relationship between job security and SEI among Chinese as well as Pakistani aspirants.
**H7.** Perceived entrepreneurial feasibility will positively mediate the relationship between intrinsic reward and SEI among Chinese as well as Pakistani aspirants.
**H8.** Perceived entrepreneurial feasibility will negatively mediate the relationship between job security and SEI among Chinese as well as Pakistani aspirants.
**H9.** Perceived entrepreneurial feasibility will positively mediate the relationship between self-efficacy and SEI among Chinese as well as Pakistani aspirants.
**H10.** The impact of (a) [environment value (ENV), intrinsic reward (INTR), extrinsic reward (EXTR), job security (JSEC), and general self-efficacy (GSE)] on TPBs indicators (ATSE, PED, and PEF) and (b) the extents of TPB on SEI will likely be different between the two countries (i.e., China and Pakistan).

## Methodological Approach

### Data Sources and Measurements

According to a study by Pizzi et al. (2020), currently, the highest proportion of youth bulge is significantly affecting the structure of the global economy in the coming years and catalyzes the achievement if properly educated and used the SDGs. Thus, data from the Efficiency-Driven Economy (China) and factor-driven economy (Pakistan) using a quantitative design *via* an anonymous questionnaire were randomly selected from eight universities out of four university participants enrolled from China and four from Pakistan.

### The Consent and Ethical Consideration of the Participants

Personal ethics are an important challenge for social scientists, and the human rights of the interviewers must be respected by advice and guidelines on survey instruments and considerations, such as securing permission and protecting anonymity and confidentiality. As a result, all qualified survey respondents were told about the intent of the study, the right to participate willingly, and the right to withdraw at any time, without explanation before data collection. Respondents were told that the information gathered will be kept private and that the research will be conducted solely for scholarly purposes. The consent form is included in the scale of the survey plan in written form. In the process of quantitative data collection, the researchers shared the purpose and objectives of the study with the participants in two languages (Chinese and English). At the same time, it was approved by the research ethics committee (School of Economics, Sichuan Agricultural University, Chengdu).

The development of constructs was driven by critical observations from previous literature, supplemented by expert opinions. A two-step procedure validated the appropriateness and logic of the constructs until performing an individual field survey. First, to ensure relevant evidence and back-projected frameworks of technological terminology, more than three field experts, tested the constructs, working on sustainability attitude, sustainable business growth, TPB, and job values. Next, to finalize the actual data collection questionnaire, the pretesting questionnaire was done on 50 students from six different universities. Then, the questionnaire, study title, research goals, demographic details about the subjects, and statements on a 5-point Likert scale (1-extremely not important/disagree, 5-extremely important/agree) were presented, accompanied by various reflecting mechanisms on the entrepreneurial intentions of the students to elicit required responses.

Finally, this research used a cross-sectional survey design to gather data; thus it was essential to investigate the existence of common method variation (CMV) to increase the effectiveness of the interviewee details (Podsakoff et al., 2012). Herman’s one-way test was used to study the CMV. According to the results of principal component analysis, the first factor explained 78.3% of the total variation. The first factor in the data can only explain 61.4% of the difference. Hence, CMV was not an issue in the current research. Furthermore, the new technique of complete collinearity proposed by Kock (2015) was used to identify the possibility of CMV scenarios. According to Kock, when the variance inflation factor (VIF) values for all constructs in the structural model are less than 3.3, it indicates that there is no collinearity. The VIF values of all constructs in this study varied between 1 and 2.843, suggesting a lack of collinearity.

### Demographic Profiles of the Participants

A total of 1,070 respondents were surveyed, and the data were gathered by handing out questionnaires and asking them to fill them out. The 15 primary agricultural disciplines offered by the universities in China and Pakistan were chosen. These courses and subjects are essential contributors to global SE; so students from these courses and subjects were chosen (Knudson et al., 2004; Wadhwa et al., 2011). As for the study of young people, the age of the respondents was limited to between > 21 and < 50. More importantly, the sampling framework includes graduate students in the respective final semesters/years of their degree programs.

The demographic evidence of the profiles of the Chinese and Pakistani respondents reveals that 48.4/55.8% were men and 51.6/44.2% were women participants, respectively. The majority of the respondents were 50.1% (*n* = 262) between 21 and 30 years of age. Predominantly, 51.7/53.3% of the students were pursuing their bachelor’s degrees and 45.3/42.8% were studying for master’s degrees, respectively. About 51.6/51.1% has shown less than 1 year of agribusiness experience. Regarding the understanding of agriculture work, 64.7/65.0% of respondents showed less than 1 year. Finally, 52.7/52.6% were inclined to have training in farming, and 47.3/47.4%, did not show any training exposure in farming with regard to entrepreneurship. Further detail of the control variables is given in Table 1.

**TABLE 1:** Demographic profiles of aspirant entrepreneurs from China vs. Pakistan.

**Group**	**China *N* = 547**			**Pakistan *N* = 523**		**Total *N* = 1070**	
	**Frequency**		**% age**	**Frequency**	**% age**	**Frequency**	**% age**
Gender	Male	265	48.4	292	55.8	557	52.1
	Female	282	51.6	231	44.2	513	47.9
	Total	547	100	523	100	1070	100
Age (years)	<21	43	7.9	41	7.8	84	7.9
	21–30	269	49.2	262	50.1	531	49.6
	31–40	181	33.1	162	31	343	32.1
	41–50	52	9.5	56	10.7	108	10.1
	>50	2	0.4	2.0	0.4	4	0.4
	Total	547	100	523	100	1070	100
Experience in agriculture	<1	354	64.7	340	65	694	64.9
	4-Jan	63	11.5	55	10.5	118	11.0
	10-May	54	9.9	52	9.9	106	9.9
	15-Nov	39	7.1	36	6.9	75	7.0
	> 15	37	6.8	40	7.6	77	7.2
	Total	547	100	523	100	1070	100
Experience in agribusiness	<1	282	51.6	267	51.1	549	51.3
	4-Jan	67	12.2	65	12.4	132	12.3
	10-May	70	12.8	67	12.8	137	12.8
	15-Nov	71	13	67	12.8	138	12.9
	>15	57	10.4	57	10.9	114	10.7
	Total	547	100	523	100	1070	100
Average monthly Income	<10,000	111	20.3	103	19.7	214	20
	10,000–15,000	272	49.7	264	50.5	536	50.1
	16,000–20,000	87	15.9	80	15.3	167	15.6
	21,000–25,000	58	10.6	57	10.9	115	10.7
	>25,000	19	3.5	19	3.6	38	3.6
	Total	547	100	523	100	1070	100
Level of education (degree)	Under graduate	283	51.7	279	53.3	562	52.5
	Master graduate	248	45.3	224	42.8	472	44.1
	Doctoral graduate	16	2.9	20	3.8	36	3.4
	Total	547	100	523	100	1070	100

### Study Measurement Scales

Firstly, this study uses the entrepreneurial intention scale provided by Liñán and Chen (2009). This structure measures key entrepreneurial intentions because these intentions best describe the entrepreneurial aspirations or the desire to own a business. Next, according to the research results scale, PED was used (Liñán and Chen, 2009); the measurement of PEF is based on the study by Krueger (1993). The scale for measuring work values builds on insights adopted from many studies (Peterman and Kennedy, 2003; Twenge et al., 2012). The scale contains the work values of four factors, namely: (1) environmental values, (2) job security, (3) intrinsic rewards, and (4) extrinsic rewards. Finally, the scale for general self-efficacy was built by taking valuable insights from the study by Gibbs (2009). This scale examined the perceived competency level of an individual by which she/he is motivated to start and pursue a business successfully.

Finally, in measuring entrepreneurial opportunity attributes, according to the specific types of entrepreneurship, the attitudes of the students toward SE and entrepreneurial goals are ranked, which provides a reasonable measurement method for SEI. In this regard, Osgood’s semantic differential scales, in essence, serve as essential measurement scales (Kriyantono, 2017). According to the choice of the respondents, the pairings with opposite characteristics in the ideal entrepreneurship were evaluated. More importantly, these characteristics pay more attention to the society and the environment and reflect SE. They think about the most vulnerable individuals in the society, global poverty, environmental issues, sustainable development, responsible use of natural resources, and maximization of social benefits, and not economic progress. The second indicator to measure the attitude toward SE requires respondents to consider the environmental and social impacts when assessing entrepreneurial opportunities. Therefore, all of these projects are based on SDGs to help understand the sustainable development intentions of youth in fostering sustainable enterprises (Org et al., 2018).

### Validity of Measurements

The validity of the scale was assessed using average variance extraction (AVE) in this analysis. The convergence efficiency is confirmed because the AVE value in this analysis is equal to or higher than 0.5, which is the threshold degree (Hair et al., 2012; Leguina, 2015). Furthermore, discriminant validity and the used heterotrait-monotrait (HTMT) criterion confirmed by measuring the association between the structures show that the approximate magnitude of discriminant validity of the inter-structure correction is less than the square of the average threshold (Henseler et al., 2016). As a result, these measurements imply that the framework is significant if the statistical analyses are necessary to arrive at a modest effects of composite reliability and cronbach’s alpha Table 2.

**TABLE 2:** Construct reliability and validity of China vs. Pakistan.

**Construct**	**Total Sample**				**China**				**Pakistan**			
	**α**	**rho_A**	**CR**	**AVE**	**α**	**rho_A**	**CR**	**AVE**	**α**	**rho_A**	**CR**	**AVE**
ATSE	0.81	0.82	0.87	0.57	0.81	0.83	0.87	0.58	0.79	0.80	0.86	0.55
ENV	0.84	0.85	0.89	0.68	0.85	0.86	0.90	0.69	0.81	0.81	0.87	0.64
EXTR	0.81	0.84	0.86	0.56	0.81	0.84	0.86	0.56	0.81	0.85	0.87	0.58
GSE	0.80	0.81	0.87	0.63	0.77	0.79	0.85	0.59	0.82	0.83	0.88	0.66
INTR	0.86	0.86	0.90	0.64	0.86	0.86	0.90	0.63	0.86	0.86	0.90	0.65
JSEC	0.75	0.77	0.84	0.57	0.74	0.76	0.84	0.57	0.74	0.77	0.83	0.56
PED	0.65	0.68	0.79	0.50	0.65	0.70	0.79	0.50	0.61	0.62	0.77	0.46
PEF	0.74	0.75	0.83	0.49	0.69	0.73	0.80	0.45	0.77	0.78	0.85	0.53
SEI	0.89	0.90	0.92	0.70	0.89	0.90	0.92	0.69	0.89	0.89	0.92	0.69

*α, Cronbach’s Alpha; (rho_A) Reliability Coefficient CR, Composite Reliability; AVE, Average variance extracted.*

### Data Scrutiny and Analysis

SmartPLS 3.0 software is used to analyze the results. The PLS is a second-generation multivariate analysis approach based on structural equations that eliminate the distribution hypothesis and have high statistical potential even for small sample research (Ringle et al., 2020). The validity of the elements used in the system can be conveniently checked using partial least squares by simplifying and validating the procedure before establishing the final structure model in each manifest variable (MV). For this purpose, the following parameters of a given tangent point are observed to test the quality and reliability of the structure used: (a) manifest variable loading (MV) > 0.50; (b) AVE > 0.5; (c) items per construct reliability > 0.5; and (d) Cronbach’s Alpha (A) > 0.70 (Henseler et al., 2016; Ringle et al., 2020). Also, the Breusch Pagan test is used to test the heteroscedasticity of the observation and confirm that there are no heteroscedasticity data results.

## Study Results

### Model Measurement Index

Model fit indices were used to verify the overall fit index of the model before checking the analysis hypotheses. Consequently, all the values of the indices for the final proposed model indicated a good match due to the PLS results of the three models (full, and split), and were evaluated using standardized root mean square residual (SRMR) (Hu and Bentler, 1998). An SRMR score less than 0.08 suggests an adequate match. The SRMR value for the entire model was 0.07 for the Chinese group and 0.06 for the Pakistani group, all of which were less than the required threshold of 0.08, suggesting a satisfactory match between the empirical and theoretical covariance matrices indicated by the models. The findings of predictive indices are shown in Table 3.

**TABLE 3:** Measurements of the model-fit-structural index.

**The goodness of fit measures**	**Estimated model**	**Total**	**China**	**Pakistan**
SRMR	<0.08	0.07	0.07	0.06
d_ULS	<95%	95%	95%	95%
d_G	<95%	95%	95%	95%
Chi-Square	8412.67	7843.51	4298.52	5080.47
NFI	>0.90	0.95	0.93	0.94

*Hu and Bentler (1998).*

**FIGURE 2:**
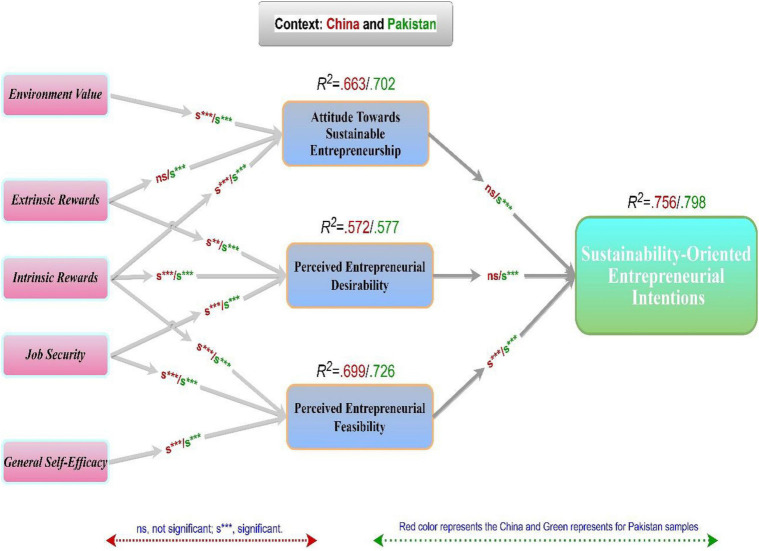
Results of the structural model linking work values and TPB to SEI.

The partial least squares structural equation modeling (PLS-SEM) analysis considered the relationship value and significance level, and the variance (R^2^) was explained (Henseler et al., 2016). To test the hypotheses of H1–H9, bootstrapping was used in 5,000 re-samples of each country, such as China and Pakistan. This provides the knowledge of the importance of the predictable relationships between sustainability-driven indicators, like ENV, EXTR; INTR; GSE; and JSEC, on the dimensions of TPB and SEIs. The results for the significance of each anticipated constructs factor loadings are shown in Table 4 and Figure 2. The total variance of the two countries is *R*^2^ = 78.3%. Moreover, for the sample of Chinese students, R^2^ explained 75.6% of the variance in SEIs. However, there are three antecedents (ATSE = 66.1%, PED = 57.5 %, and PEF = 69.9%). Overall, the Pakistani student sample, *R*^2^ = 79.8% of the variances in SEIs and the three dimensions (ATSE = 70.2 %, PED = 59.1%, and PEF = 73.1%) are explained.

**TABLE 4:** Measurement model factor loadings of China vs. Pakistan.

**Constructs**	**Items**	**Total Sample**		**China**		**Pakistan**	
		**Loadings**	**VIF**	**Loadings**	**VIF**	**Loadings**	**VIF**
ATSE	ATSE1	0.77	1.67	0.81	1.86	0.69	1.40
	ATSE2	0.79	1.83	0.80	1.93	0.73	1.64
	ATSE3	0.78	1.67	0.77	1.63	0.77	1.70
	ATSE4	0.67	1.40	0.61	1.31	0.75	1.55
	ATSE5	0.78	1.59	0.78	1.62	0.75	1.49
ENV	ENV1	0.86	2.14	0.86	2.22	0.85	1.88
	ENV2	0.83	1.91	0.84	1.96	0.80	1.74
	ENV3	0.77	1.62	0.76	1.68	0.74	1.44
	ENV5	0.83	1.88	0.84	1.95	0.80	1.68
EXTR	EXTR1	0.82	2.66	0.80	2.54	0.87	2.05
	EXTR2	0.85	2.90	0.84	2.95	0.87	2.84
	EXTR3	0.82	2.16	0.80	2.11	0.86	2.43
	EXTR4	0.69	1.22	0.70	1.19	0.67	1.35
	EXTR5	0.53	1.25	0.58	1.30	0.47	1.22
GSE	GSE1	0.79	1.57	0.78	1.52	0.78	1.58
	GSE2	0.67	1.30	0.62	1.22	0.74	1.43
	GSE3	0.83	2.04	0.81	1.79	0.86	2.51
	GSE4	0.86	2.18	0.85	1.96	0.86	2.54
INTR	INTR1	0.81	2.33	0.80	2.09	0.85	2.97
	INTR2	0.78	2.22	0.77	2.05	0.81	2.70
	INTR3	0.86	2.66	0.84	2.37	0.89	2.52
	INTR4	0.73	1.63	0.74	1.73	0.70	1.49
	INTR5	0.81	1.94	0.83	2.18	0.75	1.62
JSEC	JSEC1	0.80	1.57	0.79	1.54	0.81	1.55
	JSEC2	0.72	1.41	0.75	1.47	0.64	1.27
	JSEC3	0.68	1.29	0.66	1.25	0.69	1.32
	JSEC4	0.82	1.53	0.80	1.49	0.83	1.54
PED	PED1	0.70	1.33	0.67	1.37	0.71	1.23
	PED2	0.79	1.58	0.83	1.85	0.70	1.26
	PED3	0.49	1.09	0.44	1.06	0.58	1.13
	PED4	0.79	1.41	0.82	1.55	0.72	1.24
PEF	PEF1	0.80	1.61	0.83	1.63	0.76	1.56
	PEF2	0.70	1.36	0.70	1.33	0.69	1.40
	PEF3	0.72	1.44	0.71	1.40	0.74	1.47
	PEF4	0.60	1.22	0.44	1.12	0.73	1.44
	PEF5	0.66	1.28	0.62	1.22	0.70	1.41
SEI	SEI1	0.88	2.84	0.89	2.86	0.86	2.68
	SEI2	0.89	3.33	0.87	2.89	0.91	2.56
	SEI3	0.88	3.24	0.87	2.89	0.89	2.16
	SEI4	0.75	1.70	0.77	1.77	0.75	1.63
	SEI5	0.76	1.70	0.76	1.74	0.74	1.56

In both countries, all three TPB mediational aspects reveal a positive significance level for explaining SEI. The primary goal of the study is to determine the gaps between the subsamples of the two countries. There are essential variations in the variation described by the entire model alone or together between the two countries studied. As a result, the variance R^2^ value of the TPB in the study of Pakistani graduates is higher than in the model of Chinese graduates, indicating that TPB is more predictive power in the developing country than in the emerging economy. As a result, graduates from the developing-country have a more optimistic perspective on becoming sustainable entrepreneurs than scholars from an emerging country.

### Convergent Validity

This study examined the convergence validity of all the constructs using a benchmark test. Model discriminant validity which indicates the extent to which each construct possesses a unique attribute that makes it different from others in the conceptual model was assessed by a two-step approach. The first approach (the Fornell–Larcker criteria) included comparing the square root of AVE for each latent variable with the correlation of other constructs in the model. The second approach (HTMT) included measuring the ratio of construct correlations to within construct correlations (Henseler et al., 2016). Tables 5, 6 determine that the discriminant validity for the full and split dataset is established. Particularly, the diagonal values (Table 5) for all the constructs are greater than other values, which are provided in rows and columns (Fornell and Larcker, 1981) and HTMT values (Table 6) for all the constructs are less than 0.90 in HTMT (Voorhees et al., 2016).

**TABLE 5:** Discriminant validity of the total sample (Fronell–Larcker criterion).

**Constructs**	**ATSE**	**ENV**	**EXTR**	**GSE**	**INTR**	**JSEC**	**PED**	**PEF**	**SEI**
ATSE	***0.76***								
ENV	0.73	***0.82***							
EXTR	0.47	0.42	***0.75***						
GSE	0.71	0.79	0.52	***0.79***					
INTR	0.75	0.81	0.57	0.78	***0.80***				
JSEC	0.78	0.73	0.50	0.76	0.74	***0.76***			
PED	0.68	0.73	0.42	0.68	0.68	0.73	***0.71***		
PEF	0.75	0.77	0.51	0.78	0.80	0.78	0.71	***0.70***	
SEI	0.78	0.83	0.54	0.81	0.87	0.77	0.70	0.81	***0.84***

*Diagonal bolded and Italic values show the square root of AVE.*

**TABLE 6:** Discriminant validity of the total sample (HTMT 0.90 Criteria).

**Constructs**	**ATSE**	**ENV**	**EXTR**	**GSE**	**INTR**	**JSEC**	**PED**	**PEF**	**SEI**
ATSE									
ENV	0.87								
EXTR	1.00	0.94							
GSE	0.94	0.99	1.06						
INTR	0.92	0.97	0.99	1.00					
JSEC	0.60	0.65	0.66	0.68	0.71				
PED	0.81	0.78	0.80	0.77	0.82	0.83			
PEF	0.89	0.95	0.94	0.95	0.97	0.62	0.80		
SEI	0.90	0.93	0.93	0.92	0.95	0.70	0.85	0.98	

### Hypotheses Testing in Structural Model

To assess the proposed relationships (direct and indirect) between structures based on the structural model, PLS-SEM was used. The results that demonstrate explicitly significant positive associations, represented in Figure 2, are as follows: First, aligned with TPB, the results show that ATSE entirely drives SEI, PED, and PEF of the aspirants. Therefore, the results fully support the direct relationship between these three mediators (ATSE, PED, and PEF) and SEI. These findings are consistent with the previous study by Urban (2019) and strengthen the understanding that SEI is more complex than linear connection.

The study outcomes exemplify that extrinsic rewards and intrinsic rewards revealed a direct and positive PED impact. In contrast, job security has an inverse effect on it in the samples of both countries. Therefore, these outcomes align with the existing literature on individual entrepreneurial intentions or acting entrepreneurship tends to appreciate the power, independence, risk tanking, and innovativeness (Kirkley, 2016; Jones and Barnir, 2019). Likewise, results also support the direct and indirect association of intrinsic reward and Self-efficacy, whereas job security portrays significant negative impacts on PEF in the samples of China and Pakistan. The evidence backs up these results on self-efficacy and its positive relationship with entrepreneurial intentions (Schlaegel and Koenig, 2014), in which self-efficacy is identified as a driving force behind sustainability intentions. Because of the influence of job security in sustainable venture adoption, the findings show that it has a negative relationship with PED and that job security negatively impacts the antecedent of sustainable venture adoption. Such results are also consistent with previous research on entrepreneurial intentions, demonstrating that people concerned about their job security are less likely to start a sustainable business than those who place a higher priority on creativity and autonomy (Fitzsimmons and Douglas, 2011; Patzelt and Shepherd, 2016; Douglas et al., 2020; Sher et al., 2020).

Similarly, the findings support a direct correlation between environmental gain, intrinsic reward, and ATSE, showing that extrinsic reward has little effect on the latter. According to this research, the perception of an individual on environmental value creation significantly impacts their willingness to promote sustainable projects. These results are backed up by a study by Lyons et al. (2010) and Shepherd (2015) regarding the individual environmental value function and the implementation of sustainable business models. Furthermore, the results show a non-significant relationship between extrinsic compensation and attitudes toward SE adoption; in terms of the positive critical relationship between intrinsic reward and a theory of sustainable entrepreneurship, the study is in line with the current literature (Muñoz and Cohen, 2018; Domurath et al., 2020; Sher et al., 2020). This suggests a sustainable relationship between creativity and innovation and environmental and social value development that intrinsic reward positively affects attitudes toward sustainability (Sher et al., 2020; Shukla, 2021). The relevant findings of China vs. Pakistan samples are shown in Figure 2 and Table 7.

**TABLE 7:** Outcomes of direct effects of path coefficients among the constructs.

**Paths**	**Total sample**		**China**		**Pakistan**	
	**β-coefficient**	***t*-values**	**β-coefficient**	***t*-values**	**β-coefficient**	***t*-values**
ATSE - > SEI	0.34	12.84	0.32	9.68	0.36	8.71
PED - > SEI	0.14	5.03	0.12	3.55	0.19	4.79
PEF - > SEI	0.46	15.29	0.51	13.27	0.37	7.78
ENV - > ATSE	0.38	9.34	0.36	6.84	0.37	6.34
EXTR - > ATSE	0.08	3.08	0.06	1.73	0.15	4.14
INTR - > ATSE	0.40	9.51	0.42	7.73	0.38	6.87
EXTR - > PED	–0.01	0.38	–0.08	2.24	0.17	4.62
INTR - > PED	0.31	8.08	0.36	6.82	0.19	4.64
JSEC - > PED	0.50	14.05	0.45	8.85	0.54	13.08
INTR - > PEF	0.37	13.37	0.44	10.31	0.32	8.57
GSE - > PEF	0.29	10.07	0.24	5.26	0.29	7.44
JSEC - > PEF	0.27	8.71	0.25	6.17	0.35	9.11

*Significant standards: t-value > 1.96.*

### Mediational Coefficient Analysis

Partial least squares structural equation modeling was used to assess the mediation results of proposed interactions. The protocol for mediation research proposed by Henseler et al. (2016) and Carrión et al. (2017) was used to test the hypotheses that ATSE, PEF, and PED play a mediating role in work values, job security, self-efficacy, and SEI. Furthermore, a bootstrapping technique was used as an inferential metric to measure the *t*-values for calculating the importance of proposed mediating factors. For the usable study sample, 5,000 sub-samples were produced (with replacement) during the bootstrapping process. As a result, the importance of mediating variables was calculated using bootstrapping inferential statistics on these subsamples. In path analysis, mediational refers to the indirect influence of the dependent variable that flows through one or more mediator variables (Edwards and Lambert, 2007).

**FIGURE 3:**
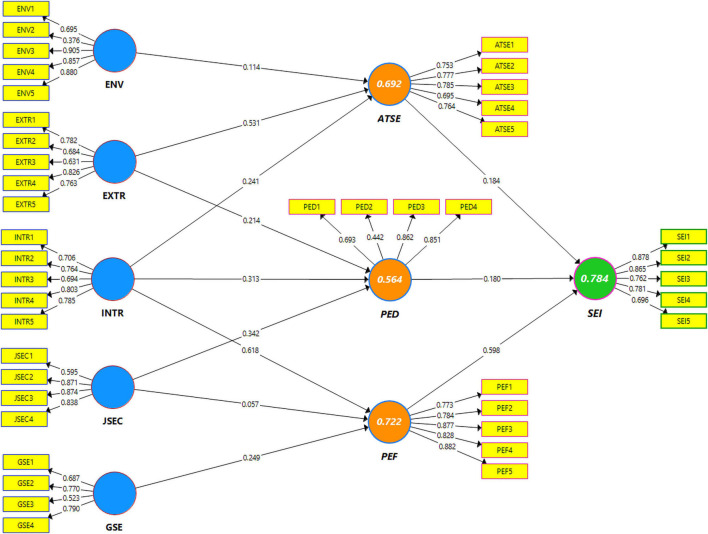
Path coefficients and factor loadings of total sample model.

According to the findings, PED thoroughly mediates the suggested paths between intrinsic rewards, extrinsic rewards, work stability, and SEI in Pakistan. In comparison, PED mediates the path between intrinsic rewards, job security, and SEI in China. The results indicate that extrinsic and intrinsic rewards are directly positive, while job security directly has significant impacts on SEI among both the country samples. These results confirm that intrinsic reward has the maximum indirect positive impact on the SEI and PEF in China. At the same time, job security shows the highest indirect positive effects on SEI and ATSE in Pakistan, which positively complements one work value creation into SEI. Hence, ATSE completely mediates the relationship between environmental value, intrinsic reward, and SEI. However, it also moderates the specific indirect positive connection between ENV and SEI; therefore, the proposed hypotheses (H1, H2, and H3) are accepted in the full and split data sets depicted in the Table 8 and Figure 3.

**TABLE 8:** Proposed hypotheses mediational effects among constructs of China vs. Pakistan.

**Paths**	**Total Sample**		**China**		**Pakistan**	
	**β-coefficient**	***t*-values**	**β-coefficient**	***t*-values**	**β-coefficient**	***t*-values**
ENV - > ATSE - > SEI	0.13	7.27	0.11	5.43	0.13	4.89
EXTR - > ATSE - > SEI	0.03	2.92	0.02	1.67	0.05	3.50
INTR - > ATSE - > SEI	0.14	7.59	0.14	5.86	0.14	5.52
EXTR - > PED - > SEI	0.00	0.37	–0.01	1.81	0.03	3.29
INTR - > PED - > SEI	0.04	3.69	0.04	2.73	0.04	2.86
JSEC - > PED - > SEI	0.07	5.14	0.05	3.52	0.10	4.82
GSE - > PEF - > SEI	0.13	7.45	0.12	4.76	0.11	5.16
INTR - > PEF - > SEI	0.17	9.65	0.22	8.03	0.12	5.29
JSEC - > PEF - > SEI	0.14	8.53	0.13	5.58	0.13	6.45

*Significant standards: t-value > 1.96.*

Consequently, H4, H5, and H6 are accepted, showing a significant relation with the mediational variables. The study finding entitles that ATSE translates environment value and intrinsic reward toward SEI. Thus, the higher is the motivation and preference for environment value and intrinsic reward, the higher is the SEI and vice versa with extrinsic rewards. Further, from the results, environment value has the second-highest indirect positive impact on SEI.

**FIGURE 4:**
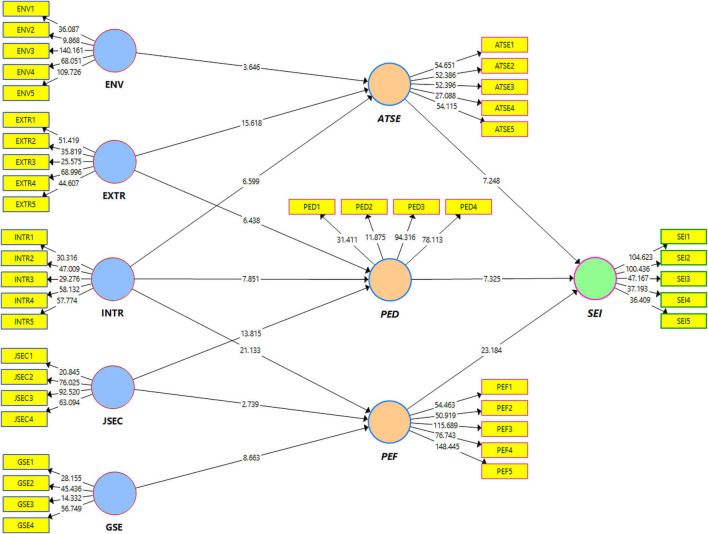
Direct and indirect significant relationships of total sample model.

Similarly, the results show that PEF mediates the path relations among intrinsic reward, job security, and general self-efficacy for testing the H7, H8, H9, and SEI and designates that GSE and INTR show an indirect and positive influence on SEI. Therefore, the findings reveal that intrinsic reward and job security meet the mediational criterion developed by Baron and Ensley (2006), which illustrates that the antecedent of the predicted variable must be significantly related to the mediator. H9 is also accepted as these constructs, i.e., intrinsic rewards, job security, and general self-efficacy satisfy the mediational criterion in the samples of both countries. These worth noting findings from the models of both the countries are to be discussed furthermore and are shown in Table 9 and Figure 4.

**TABLE 9:** Total indirect effects among the constructs of China vs. Pakistan.

**Paths**	**Total Sample**		**China**	**Pakistan**		
	**β-coefficient**	***t*-values**	**β-coefficient**	***t*-values**	**β-coefficient**	***t*-values**
ENV - > SEI	0.128	7.27	0.114	5.432	0.132	4.892
EXTR - > SEI	0.025	2.464	0.01	0.756	0.085	5.005
GSE - > SEI	0.125	7.447	0.122	4.755	0.108	5.16
INTR - > SEI	0.351	15.735	0.402	12.806	0.292	10.416
JSEC - > SEI	0.206	11.768	0.181	7.421	0.233	10.643

*Significant standards: t > 1.96.*

### Measurement Invariance

The Measurement Invariance process is required prior to conducting multi-group analysis before comparing the findings of Chinese and Pakistani samples. The main purpose of this test is to ensure that both groups interpret the measures in the same way. The measurement invariance of composite model (MICOM) methods are based on the latent variable scores, which represent these latent variables as composites, which are linear combinations of indicators, and the variable weights are calculated using the PLS-SEM technique (Henseler et al., 2016). This research used the MICOM (Henseler et al., 2016). The process of MICOM has the following three stages: (a) configure invariance assessment (the measurement models of both the groups have the same fundamental component structure); (b) compositional invariance assessment (composite scores are not substantially different between groups); and (c) equality of composite means, values, and variances. If customizable and compositional variances are established, partial measurement invariance is verified, and the path coefficient may be compared between the two groups. Full measurement invariance is proven if partial measurement invariance is demonstrated and the composite has identical mean values and variance across all groups. The PLS-algorithm and PLS-permutation procedures, with a 5,000 sub-sample and a two-tailed test, were performed. The procedure compared the original score correlations (c) with the correlations obtained from the empirical distribution after running the permutation process (cu); compositional invariance is defined if c approaches the 5 percent quantile of cu (Henseler et al., 2016).

### Multigroup Analysis

Finally, this paper also presented the PLS-Multigroup analysis (MGA) results, representing the differences between the path comparisons of China vs. Pakistan. The MGA method was used to compare the statistical differences between the two countries. For this purpose, we have devised (Henseler et al., 2009) a nonparametric PLS-MGA model approach to inspect the H10 results if the comparative relationship among work values, TPB magnitudes, and SEIs are likely to differ across the two countries in this study. However, the MGA results only differentiated the paths of the two countries with regard to JSEC-PED and INTR-PED (significantly). However, the results of MGA did not demonstrate the significant differences between both countries. According to the MICOM method, there is no need to proceed to the next stage if the compositional variance is not discovered; nevertheless, to give readers a better understanding, this research did perform the MGA, despite the fact that MGA is not relevant without verifying compositional invariance.

Thus Tables 10, 11 indicate that no partial measurement invariance for the two connections that were determined to be significant at *p*-value as the difference of group-specific route coefficients is either < 0.05 or > 0.95 (Henseler et al., 2009) indicating that compositional invariance was not discovered in the paths of the two countries, comparatively. Other routes were similar in both the countries, while the italic *p*-values indicate that significant differences between the two economies partially support H10.

**TABLE 10:** Compositional invariance of China vs. Pakistan.

**Compositional invariance C = 1**					**Equal mean**				**Equal variance**			
**Constructs**	**Configure invariance**	**C = 1**	**P- Mean**	**5% c**	**Partial MI established**	**Mean difference**	**2.50%**	**97.50%**	**Variances difference**	**2.50%**	**97.50%**	**Full MI established**
ATSE	Yes	0.998	1.000	0.999	No	0.421	–0.119	0.118	–0.25	–0.127	0.128	No
ENV	Yes	0.999	1.000	0.999	No	0.421	–0.12	0.121	–0.245	–0.128	0.122	No
EXTR	Yes	0.998	1.000	0.999	No	0.432	–0.123	0.123	–0.433	–0.127	0.119	No
GSE	Yes	0.997	0.999	0.997	No	0.497	–0.123	0.121	–0.243	–0.136	0.141	No
INTR	Yes	0.999	1.000	0.999	No	0.406	–0.119	0.124	–0.334	–0.124	0.125	No
JSEC	Yes	0.996	0.999	0.998	***Yes***	***0.113***	–0.117	0.119	–0.221	–0.141	0.143	Yes
PED	Yes	0.999	1.000	0.999	No	0.178	–0.123	0.12	–0.401	–0.122	0.121	No
PEF	Yes	1.000	1.000	1.000	No	0.403	–0.121	0.123	–0.127	–0.115	0.109	No
SEI	Yes	1.000	1.000	1.000	No	0.352	–0.117	0.124	–0.369	–0.125	0.125	No

*Italic and boldface values violate the measurement invariance assumptions between China and Pakistan.*

**TABLE 11:** PLS-multigroup analysis of China vs. Pakistan.

**Paths relationship**	**Path coefficients-diff (China—Pakistan)**	***p*-value (China vs. Pakistan)**
ATSE - > SEI	0.015	0.624
ENV - > ATSE	0.031	0.311
EXTR - > ATSE	0.011	0.571
EXTR - > PED	0.105	0.066
GSE - > PEF	0.006	0.552
INTR - > ATSE	0.046	0.748
***INTR - > PED***	***0.174***	***0.995***
INTR - > PEF	0.035	0.259
***JSEC - > PED***	***0.146***	***0.002***
JSEC - > PEF	0.030	0.749
PED - > SEI	0.022	0.325
PEF - > SEI	0.018	0.640

*Italic and boldface values depict significant differences between China and Pakistan.*

## Discussion

This study has many functional and theoretical ramifications that are worth noting. These findings point to the factors that drive a plethora of sustainable start-up opportunities and entrepreneurship growth among aspirant entrepreneurs in China and Pakistan. First, the hypotheses (H1, H2, and H3) proposed that ATSE has a mediational relationship among work values, namely, environmental value, intrinsic reward, extrinsic reward, and SEI. The findings revealed that ATSE thoroughly mediates the significant positive relationship between ENV and INTR, but has a significant negative impact of EXTR on SEI in the samples of both countries can be seen in Table 8. PLS-SEM bootstrapping revealed that these hypothesized direct and indirect effects for full and split data set are highly significant. The intentions of the individuals to pledge sustainable ventures in China and Pakistan are positively correlated with work value creation, environmental values, and intrinsic rewards predicted certain behaviors and desires for sustainable businesses in both countries. Regarding the outcomes on the intrinsic and extrinsic rewards, the results of the current study are supported by the work of Short and Hawley (2015), Kirkley (2016), Sargani et al. (2020), and Sher et al. (2020), encouraging the effect of intrinsic and extrinsic rewards in the uptake of sustainable ventures in the United States, New Zealand, Pakistan, and China, which was accompanied by the substantial and encouraging effect of intrinsic and extrinsic rewards in the uptake of sustainable ventures.

These findings indicated that the more the aspirant entrepreneurs value hegemony, independence, risk-taking, and ingenuity, the more likely they become future sustainable magnates. In terms of the inverse function of work security, the pragmatic analysis (Patzelt and Shepherd, 2016) based on graduate alumni of Australian universities from the United States, Europe, India, and China with relative comprehension indices, show that the individuals have lower long-term market ambitions and higher job stability. As a result, in contrast to intrinsic and extrinsic rewards, individuals who prioritize career stability and job longevity have lower long-term market uptake intentions than people who value creativity and freedom (Twenge et al., 2012; Sher et al., 2020).

In addition to the H4, H5, and H6, the PED in China and Pakistan indicates a mediational interaction among intrinsic reward, extrinsic reward, and job security, on SEI. The findings support theories for environmental value and intrinsic reward in terms of the mediational function of PED. In the samples of both countries, the bootstrapping study revealed that PED significantly mediates the relationship between extrinsic reward, intrinsic reward, and job security on SEI (Fitzsimmons and Douglas, 2011; Sher et al., 2020). Similarly, extrinsic rewards show a meaningful relationship with PED in full and split datasets of the model. Also, in the study of Baron and Ensley (2006), the PED reached the mediational targets. However, in our study, the intrinsic reward revealed a higher direct and indirect impact on SEI of both samples shown in Table 9.

These results are consistent with those of Liñán and Fayolle (2015) and Sher et al. (2020), who investigated the effects of workable values, inspiration, and intentions on SEI Spain, based on an analysis (Majid and Koe, 2012; Koe et al., 2015; Sher et al., 2020) in Malaysia and Pakistan, respectively, showing that intrinsic incentive and ATSE are the predominant motivators for pursuing the sustainable enterprise. Similarly, (Fitzsimmons and Douglas, 2011; Sargani et al., 2020) in India, China, Australia, and Thailand, a comparative analysis of aspiring entrepreneurs was conducted and pointed out that attitudes of the people toward sustainability and their job beliefs are essential considerations in determining potential venture adoptions. The work of Sargani et al. (2020) and Sher et al. (2020) found that positive actions, a sustainable mindset, and entrepreneurial viability are guiding factors in sustainable intentions and uptake of sustainable performing projects, which corroborate the results of this report. In a related way, these results are consistent with the findings of Shepherd and Patzelt (2014), Domurath and Patzelt (2019), Sargani et al. (2020), and Sher et al. (2020), with regard to the connections between environmental value and long-term sustainable goals. These findings suggest that a higher degree of ecological benefit and intrinsic compensation is linked to a more optimistic outlook toward implementing sustainable enterprises.

The current study on the hypotheses of China vs. Pakistan (H7; H8; H9) confirmed that PEF mediates between intrinsic rewards, job security, and self-efficacy, on SEI, in both countries. The findings endorsed all independent connections on SEI interactions, such as intrinsic reward, job stability, and self-efficacy. Whereas in China, the intrinsic reward had a higher indirect impact, and job security had a more substantial indirect influence on PEF, resulting in it being a center of mediation due to its capacity to meet the preconditions of mediational research (Baron and Ensley, 2006; Carrión et al., 2017). The bootstrapping study shows that the PEF has significant mediating effects between intrinsic rewards, work safety, self-efficacy, and SEI in the samples of both countries. Prior literature on sustainability goals provides sufficient evidence for the importance of job security and general self-efficacy in this research (Chen et al., 2001; Shepherd and Patzelt, 2014; Diallo, 2019; Sargani et al., 2020). As a result, the results of the study support the idea that the greater is the self-efficacy of a potential entrepreneur, the more likely they are to have sustainable start-up business plans.

Finally, this paper presented the PLS-MGA model approach to check the results of H10 if the comparative relationship between work values, TPB magnitudes, and SEIs are likely to differ across countries. Thus Tables 10, 11 show that there was no measurement invariance determined to be significant at *p*-value, for the difference of group-specific route coefficient are either < 0.05 or > 0.95 (Henseler et al., 2009) indicating that compositional invariance was not found in the paths of the two countries, comparatively, whereas the italic *p*-values indicate that significant differences between the two economies partially support H10.

### Sustainability-Oriented Model From a Theoretical Perspective

The research provides a deeper explanation of the applicability of TPB in SEI from a theoretical standpoint. A development to estimate SEI is suggested, and it can also be applied to another stereotype-specific entrepreneurship (Schaefer et al., 2015). The study adds to the existing literature on entrepreneurship by revealing how different entrepreneurship aim models can be tailored to suit the specific context (Shepherd and Patzelt, 2014). This study added to the SEI literature by creating a symbolic structure for long-term job value and testing it in two countries (China and Pakistan). The findings suggest that using a combined model is a viable option that has gained little coverage in the literature.

The findings of this analysis strongly reinforce the notion that the TPB should be used while researching type-specific entrepreneurial intentions (Ajzen, 1991; Liñán and Chen, 2009). The findings of the report back up the findings by Liñán and Fayolle (2015), Sargani et al. (2020), and Sher et al. (2020) that recommend using aim models and demonstrate how they can be extended to different types of entrepreneurship intentions. To put it another way, it means that they adopt substantially. In a nutshell, understanding the differences in entrepreneurial opportunities is essential for implementing different entrepreneurial models. Besides, the thesis explores SEI drivers by incorporating various aim models into SE models. It broadens the range of viable alternatives and the range of options available to young people.

### Implications for Practice and Policy Based on New Insights

The paper contributes to the emerging literature on SE by including fresh policy ideas and current experiences. First, it discusses how various job principles influence the attitude of an individual toward long-term entrepreneurship in various economies. Furthermore, the prior literature by Patzelt and Shepherd (2016), Lundmark et al. (2019), and Sher et al. (2020) advocated for the internal balancing between principles, assumed skills, and motivations in constructing SE. At the same time, it resulted that the career of an individual and professional decisions, which are closely linked to workable values and that the decision to proceed on a sustainable endeavor is likely to be influenced by their expectations for the work environment (Jaén and Liñán, 2013; Hsu et al., 2017; Sher et al., 2020). The findings show that workable principles are important motivators and building blocks for a positive attitude toward sustainability and SE (Parra, 2013).

Second, the findings add to the growing body of knowledge in the field of SE about the relationship between job principles, behaviors, and intentions (Avey et al., 2010; Sher et al., 2020; Shukla, 2021). Furthermore, the findings of the study also provide helpful information on enterprise viability and desirability by advancing a substantial mediating impact on the SEI of students.

Third, the findings are similar to those of Hewlett et al. (2009) and Sher et al. (2020), which also asserted that millennials are environmentally aware and have a higher attitudinal tendency toward sustainable enterprises. They further emphasize the importance of providing young entrepreneurs with benefits rather than cash rewards for their start-workups.

Finally, as a result, the environmental enterprise will become a central motivating force for our shared future sustainability. In both economies, our mutual future market prosperity is a vital driving force in leapfrogging the degree of SEI influence. The SE progressive management approach will create new business systems, markets, and services in creating new goods and services. In the enterprise process, the SE innovative management approach will generate new goods and services and increase the impact of social and environmental business operations in the future.

## Conclusion and Policy Implications

All in all, this research has made a significant contribution to the literature on SEIs by creating an expanded framework of TPB that incorporates the function of individual work values, and by testing this model in two different nations (China and Pakistan). The findings indicated that there was support for this integrated model approach, which had received little attention or emphasis in the literature up to this point. Students from both countries are expected to launch their own sustainable businesses. However, the comparatively stronger intentions are shown by Pakistani students. In the context of the sustainability values disposition, this study has contributed toward theoretical underpinning for suggesting a new understanding of outlook by including work values (i.e., ENV, JSEC, GSE, INTR, and EXTR (rewards) along with the meditational role of three attitudinal magnitudes of TPBs though comparing two economies (i.e., developing and emerging). The results found the stronger role of work values of sustainable business inclinations and its effect on attitudinal dimensional among students from a developing country; these variables explain the impacts of specific country context. The results advocate that students from both countries show positive sustainable oriented intentions toward self-employment and sustainable enterprises. However, this study only compares one efficiency-driven economy (China) and one factor-driven developing country (Pakistan); inevitably the results are generalizable only to these countries only.

### Research and Practice Implications

This paper enriches the existing literature in three research streams. First, it enriches the literature on SEI and TPB models. Several prior studies have predicted SEI cross-culturally by exploring the role of cultural, demographic, socio-economic, and several other variables; this study contributes to the existing research by showing how work values and TPB together affect SEI). The findings related to work values make an explicit comparison to explain differences in SEIs between the two countries.

The findings of this study provide new insights into the existing literature on SE. First, by contributing to the sustainability work values that are important for developing theories related to the entrepreneurial process and behavior, how different work values drive the attitude of an individual toward the uptake of SE.

Second, this paper attempts to strengthen the role of TPB and its magnitudes. These findings highlighted the validity of the TPB model, elaborate on the mediating role of its attitudinal dimensions of TPB, and also provide a comparative understanding of TPB and its extents in each country. In addition, the findings of the study offer valuable insights on perceived venture feasibility and desirability by advancing a significant mediating effect on the SEI of the students.

Third, this study enriches the literature on an integrated model of personality and TPB by providing comparative roles of this integrated model regarding two economies (i.e., emerging and developing). The results provide an in-depth understanding of these variables and extend the existing literature with respect to sustainability work values and TPB. In addition, the findings of the study offer valuable insights on perceived venture feasibility and desirability by advancing a significant mediating effect on the SEI of the students in the two Asian countries.

### Policy Implications

This study also suggests implications for universities, institutions, and policymakers to design policies that can enhance sustainable entrepreneurial culture in both countries. Based on the research findings, the authors recommend that new regulations must be implemented to help students develop sustainable business practices. Less risk taking, more effective implementation of sustainable entrepreneurial concepts, and finding possibilities should be the educational approaches of the universities. Universities in both countries should offer new educational techniques including expert instructors and successful entrepreneurs to foster values of innovation, creativity, and risk-taking, particularly among Pakistani students. Decision makers should plan and develop policies at the government and institutional levels to foster a sustainable entrepreneurial culture by ensuring the protection of aspiring entrepreneurs. There is a need to compensate prospective entrepreneurs for their efforts in ways other than monetary compensation, which may allow the SEI to rise to a higher level in both countries.

In this manner, sustainable possibilities will increase the likelihood of sustainable start-ups and encourage SE in both economies. As a result, the research has implications for promoting sustainability-driven work values and endorses that work values should be given greater emphasis in accelerating individual attitudes toward SE. Using the practices of role models in the industry and engaging with them to create a good picture of sustainable enterprises would be one potential approach to increase the adoption of SE as a profession while also driving sustainable development.

### Future Prospects

On the other hand, sustainable agribusiness is a collection of activities and a community-wide bargaining mechanism that pushes and pulls overlapping forces to address the emotional issue of preserving food and fiber. Similarly, inspiring nonbusiness people to pursue entrepreneurship may be another way to significantly affect the extent of long-term entrepreneurial aspirations and subsequent implementation. Creating opportunities for capacity development at educational institutions, as well as fostering themes of competitive venturing within established enterprises, will prove to be tremendous feedback in encouraging such entrepreneurs across diverse countries to satisfy the need of the current society for food and fiber without jeopardizing the potential of the future generations to cope using its resources. In the end, it would take a typical mentality and wisdom to assist our society in overcoming the most difficult challenges using creative business approaches. The active participation of youth in sustainable development activities is critical to creating sustainable, equitable, and healthy economies by the target date, as well as averting the worst risks and challenges to sustainable development, such as the effects of climate change, gender disparities, war, displacement, probability, unemployment, and poverty alleviation, especially in rural settings.

### Limitations

The research still has several drawbacks, one of which opens up new possibilities for future researches. To begin with, the suggested relations in this study are context-specific, respectively, sustainable value formation and sustainable entrepreneurship, and did not consider the distinction between actual actions (reality) and purpose (perception) (Liñán and Chen, 2009). Second, a possible restriction is the sample size, which was limited to eight agrarian university students from only two Asian nations. A study providing a solid foundation for the inquiry will strengthen support for the proposed model and provide a comparative image of distinct classes of individuals, giving proper assistance to suggested ties that may be homogeneous economies. Third, gender was not included in this study; however, considering the role of gender, especially in emerging and developed economies, would strengthen the existing model. As a result, a larger-scale analysis is needed to generalize the results. Despite its shortcomings, this study offers valuable guidance to academics, policy planners, and government entities, allowing them to make informed decisions on improving and expanding SE in both economies. It should look at these links through various entrepreneurship opportunities representing different tiers and types of businesses for future generations.

## Data Availability Statement

The raw data supporting the conclusions of this article will be made available by the authors, without undue reservation.

## Ethics Statement

The studies involving human participants were reviewed and approved by the Ethics Committee College of Economics, Sichuan Agricultural University, Chengdu. The patients/participants provided their written informed consent to participate in this study.

## Author Contributions

GRS: data curation and software. GRS and AAC: formal analysis and validation. GRS, AAC, and MH: investigation, writing—review, and editing. GRS, DZ, and YJ: resources, visualization, and supervision. GRS, NK, and MH: data entry. All authors have read and approved the final version of the manuscript.

## Conflict of Interest

The authors declare that the research was conducted in the absence of any commercial or financial relationships that could be construed as a potential conflict of interest.

## Publisher’s Note

All claims expressed in this article are solely those of the authors and do not necessarily represent those of their affiliated organizations, or those of the publisher, the editors and the reviewers. Any product that may be evaluated in this article, or claim that may be made by its manufacturer, is not guaranteed or endorsed by the publisher.
